# Multi-wavelength diffractive optical neural network integrated with 2D photonic crystals for joint optical classification

**DOI:** 10.1515/nanoph-2025-0168

**Published:** 2025-07-08

**Authors:** Yuanyuan Zhang, Kuo Zhang, Pei Hu, Daxing Li, Shuai Feng

**Affiliations:** School of Science, 12505 Minzu University of China, Beijing 100081, China; State Key Laboratory of Advanced Optical Communication Systems and Networks, Department of Electronic Engineering, Shanghai Jiao Tong University, Shanghai 200240, China; College of Humanities and Law, Beijing University of Chemical Technology, Beijing 100029, China

**Keywords:** optical computing, optical machine learning, optical neural network, diffractive deep neural network, multi-wavelength parallelism, 2D photonic crystals

## Abstract

Optical neural networks (ONNs) have demonstrated unique advantages in overcoming the limitations of traditional electronic computing through their inherent physical properties, including high parallelism, ultra-wide bandwidth, and low power consumption. As a crucial implementation of ONNs, on-chip diffractive optical neural network (DONN) offers an effective solution for achieving highly integrated and energy-efficient machine learning tasks. Notably, wavelength, as a fundamental degree of freedom in optical field manipulation, exhibits multidimensional multiplexing capabilities that can significantly enhance computational parallelism. However, existing DONNs predominantly operate under single-wavelength mechanisms, limiting the computational throughput. Here, we propose a multi-wavelength visual classification architecture termed PhC-DONN, which integrates two-dimensional photonic crystal (PhC) components with diffractive computing units. The architecture comprises three functional modules: (1) a PhC convolutional layer that enables multi-wavelength feature extraction; (2) a three-stage diffraction layer performing parallel modulation of optical fields; and (3) a PhC nonlinear activation layer implementing wavelength nonlinear computation. The results demonstrate that the PhC-DONN achieves classification accuracies of 99.09 % on the MNIST dataset, 66.41 % on the CIFAR-10 dataset, and 92.25 % on KTH human action recognition. By introducing a wavelength-parallel classification mechanism, the architecture accomplishes multi-channel inference during a single light propagation pass, resulting in a 32-fold enhancement in computational throughput compared to conventional DONNs while improving classification accuracy. This work not only establishes a novel optical classification paradigm for multi-wavelength optical neural network, but also provides a viable pathway towards constructing large-scale photonic intelligence parallel processors.

## 1 Introduction

The relentless evolution of artificial intelligence has propelled deep learning models to unprecedented achievements across computer vision and natural language processing domains [1], [2], [3], [4], [5]. However, traditional electronic architectures confront dual serious constraints – the arithmetic power wall and the energy consumption wall – when processing large-scale neural networks, with their physical bottlenecks being critically exacerbated by the exponentially growing model complexity [6], [7]. Photonic computing emerges as a transformative alternative, exploiting light’s intrinsic properties of an ultralow latency, massive parallelism, and energy-efficient transmission [8], [9], [10], [11], [12]. Optical neural networks (ONNs), implemented through photonic field manipulation, have demonstrated orders-of-magnitude improvements in terms of computing efficiency compared to electronic processors for matrix operations critical to image recognition and signal processing [13], [14], [15], [16], [17]. Diverse ONN architectures have recently materialized, spanning, coherent interferometric networks using Mach–Zehnder interferometer (MZI) arrays [18], [19], [20], [21], [22], wavelength-division multiplexing (WDM) systems employing microring resonators [23], [24], [25], [26], [27], [28], [29], and phase-change material (PCM) based reconfigurable platforms [30], [31], [32]. However, these architectures are severely limited by fabrication challenges associated with further integration. Diffractive optical neural networks (DONNs), which implement optical field modulation through cascaded diffractive planes, establish a novel framework for large-scale on-chip integrated optical computing [33], [34], [35], [36], [37], [38], [39], [40], [41]. However, current DONNs are generally restricted to single-wavelength operating modes and fail to fully exploit the multi-degree-of-freedom characteristics of optical fields. This limitation fundamentally stems from the weak spectral selectivity of traditional diffractive layers: Their phase modulation capability is wavelength-dependent, yet exhibits insufficient sensitivity over broad bands. In scenarios requiring multi-wavelength parallel computation, operations are typically limited to a small number of widely separated wavelengths within a broad spectral band [42]. If DONN can be integrated with advanced wavelength-based modulation devices, where the wavelength information is pre-processed prior to the multi-wavelength light entering the DONN, it is expected to provide an opportunity to significantly enhance the parallel computational capabilities of DONN [43], [44].

Photonic crystals (PhCs), as artificial bandgap materials, enable precise manipulation of the photon local density of states (LDOS) through their periodic dielectric structures [45]. When the periodicity is intentionally disrupted in PhCs, defect modes emerge within the photonic bandgaps, thereby localizing photons at specific frequencies to achieve optical signal control with wavelength precision [46], [47], [48]. Through deliberate defect design, two-dimensional (2D) PhCs can generate high-Q resonant modes at designated wavelengths, creating an ideal platform for wavelength-selective modulation. Recent studies have demonstrated that PhC-based microcavity arrays can implement high-performance optical convolution kernels [13]. Moreover, PhCs are compatible with CMOS manufacturing processes, positioning them as ideal components for overcoming the wavelength limitations in conventional DONNs [49].

Here, we propose a photonic crystal-diffractive optical neural network (PhC-DONN) architecture for task classification by joint inference, constructed by integrating 2D PhC processing units with diffractive computing elements. It consists of a PhC convolutional layer, three diffractive layers, and a PhC nonlinear activation layer. Independent control of multi-wavelength optical signals within the PhC-DONN is realized through the design of defect structures in the PhCs and precise modulation of the refractive index of silicon pillars within these regions, thereby enhancing performance for multi-wavelength visual classification tasks. This approach implements an advanced wavelength-parallelization strategy: instead of altering weights in the diffractive fully-connected layers per wavelength channel, the front-end PhC layer generates distinct input intensity patterns for different wavelengths at the convolutional input stage. This bypasses the wavelength-dependent phase modulation limitation inherent to diffractive networks. Simulation results demonstrate that the PhC-DONN achieves superior image classification accuracy through the joint inference of optical intensities at different wavelengths, reaching 99.09 % accuracy on the MNIST dataset and 66.41 % on the CIFAR-10 dataset. For video classification, each wavelength channel corresponds to an individual video frame, enabling full-frame processing via only one light forward propagation. This approach significantly improves information throughput and inference speed, achieving a 92.25 % classification accuracy on the KTH dataset for human action recognition.

As shown in Table 1, this work demonstrates significant advantages in image and video classification through multi-wavelength parallel processing. Compared to conventional amplitude-phase-based modulation diffractive neural network schemes, our design achieves a classification accuracy of 99.09 % on the MNIST dataset, 66.41 % on the CIFAR-10 dataset, and 92.25 % on the KTH dataset, accompanied by a 32-fold increase in processing efficiency. This evolution will facilitate the integration of additional wavelength modes into photonic neural networks, thereby overcoming current limitations in parallelism and enabling more complex multitasking parallel classification scenarios. We believe that PhC-DONN provide an enhanced framework for all-optical parallel classification and video task processing.

**Table 1:** Blind testing accuracies of reported optical/photonic neural networks.

Refs.	Dimension	Function	MNIST	KTH	Parallel classification	Architecture	On-chip
**This work**	**Wavelength**	**Image/video recognition**	**99.09 %**	**92.25 %**	**Yes (32)**	**CNN(PhC)+DONN**	**Yes**
Luo [50], Light Sci. Appl.	Polarization	Image recognition	93.75 %	–	Yes (3)	D^2^NN	Yes
Zhang [37], Adv. Photonics Nexus	OAM	Image recognition	85.49 %	–	Yes (4)	D^2^NN	No
Fang [51], Light Sci. Appl.	OAM	Image recognition	97.2 %	–	No	CNN	No
Duan [42], Nanophotonics	Wavelength	Image recognition	96.5 %	–	Yes(4)	D^2^NN	No
Meng [52], Nat. Commun.	Wavelength	Image recognition	92.17 %	–	Yes(4)	CNN	Yes
Bai [53], Light Sci. Appl.	Wavelength	Image recognition	87.74 %	–	No	D^2^NN	No
Li [54], Sci. Adv.	Wavelength	Image recognition	95.05 %	–	No	D^2^NN	No
Cheng [35], Nat. Commun.	–	Multimodal recognition	85.7 %	–	No	DONN	Yes
Fu [34], Nat. Commun.	–	Image recognition	90.0 %	–	No	DONN	Yes
Lin [55], Science	–	Image recognition	93.39 %	–	No	D^2^NN	No
Zhou [41], Nat. Photonics	–	Image/video recognition	96.00 %	90.50 %	No	D^2^NN	No

## 2 Methods

### 2.1 PhC-DONN architecture

**Figure 1:**
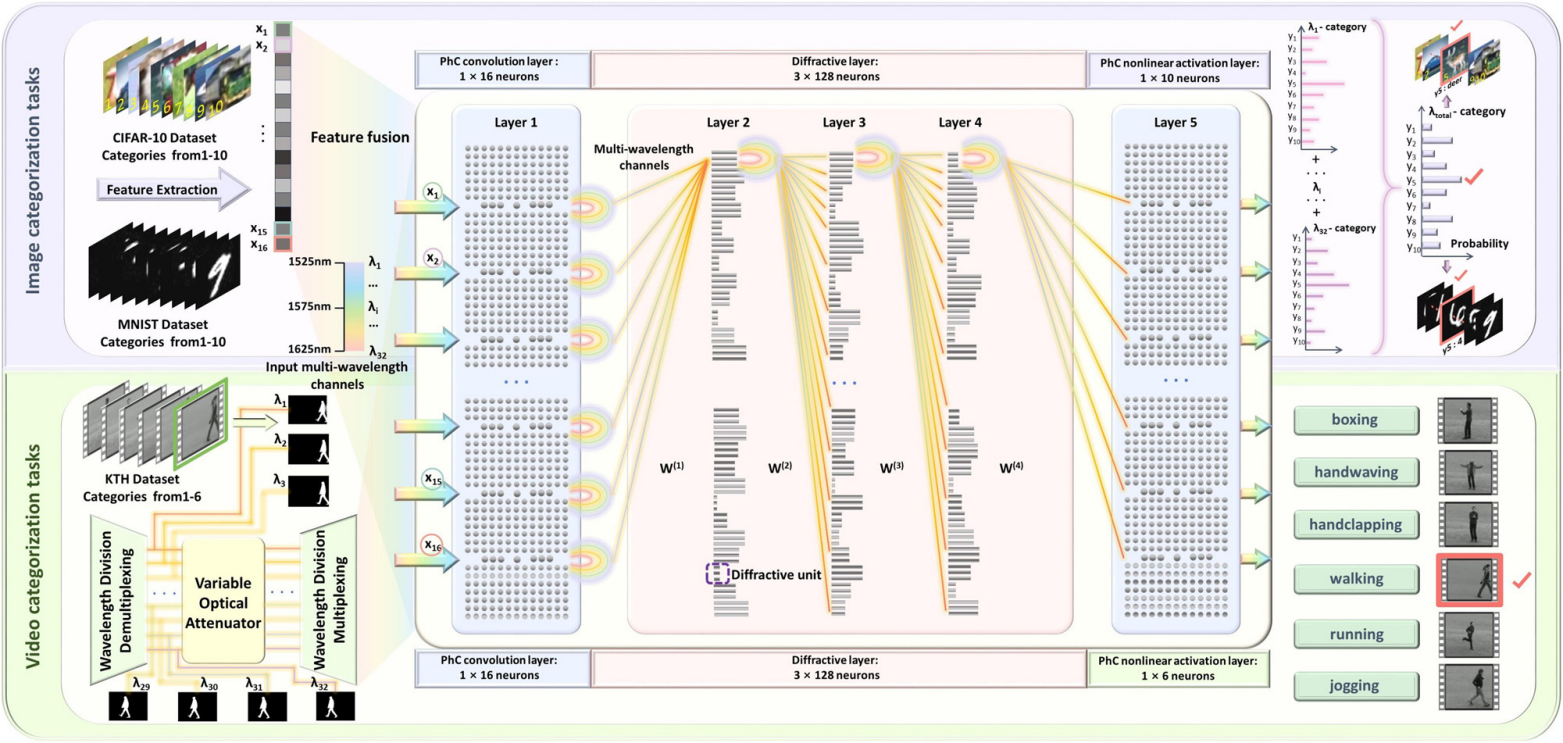
Schematic diagram of the PhC-DONN architecture. This optical neural network is designed for multimodal visual classification tasks. It comprises a PhC convolutional layer, three diffractive layers, and a PHC-based nonlinear activation layer, capable of processing image and video data for classification. In this architecture, each PhC unit is the fundamental building block, enabling neural network computation through precisely controlled multi-wavelength optical signal propagation and collaborative processing with diffractive units (DUs).

We present a multi-wavelength-based DONN for visual classification, as illustrated in Figure 1. The architecture is constructed with a PhC convolutional layer, three cascaded diffractive layers, and a PhC-based nonlinear activation layer. In the image classification task, the input data is transformed into feature vectors through feature extraction and fusion, which are subsequently mapped onto the input layer of the PhC-DONN. Each feature component is loaded with equal intensity into 32 groups of optical signals with distinct wavelengths. The PhC convolutional layer consists of 16 PhC units. The system adopts a multi-wavelength encoded input method, receiving 32 groups of optical signals with distinct wavelengths, and regulates the transmittance of the corresponding wavelengths through the PhC convolutional layer. This mechanism enables independent encoding and efficient transmission of wavelength-specific signals to the diffractive layers. In the diffractive layers, optical signals undergo optical computation through three diffractive layers. Each diffractive layer contains 128 diffractive units (DUs), with each unit composed of three Si grooves filled with SiO_2_. Each DU performs fixed-weight operations by adjusting the diffraction effects of light waves through phase modulation, thereby implementing linear transformations in the neural network. In the PhC nonlinear activation layer, optical signals at different wavelengths generate distinct transmittance responses due to variations in the refractive index of PhC defects, completing the nonlinear computation of the PhC-DONN. Both the PhC convolutional layer and the PhC nonlinear activation layer utilize PhC units. In experiments, the refractive index of each PhC unit is precisely controlled by micro-heaters, where an applied voltage adjusts the local temperature to alter optical transmittance, ultimately achieving dynamically programmable computing. The PhC-DONN performs joint inference on the intensities of all 32 wavelength groups, with the optical power at each PhC output port corresponding to the probability distribution of classification results. Finally, the output port with the highest total power across all wavelengths determines the predicted category.

For video classification tasks, the video data are initially decomposed into 32 frames. Each frame is loaded onto 32 demultiplexed (DEMUX) wavelength channels with distinct wavelengths. After passing through the PhC convolutional layer, optical signals corresponding to each frame are independently modulated and encoded by adjusting the transmittance of the PhC units at their respective wavelengths. The diffractive layers and the PhC nonlinear activation layer then perform optical computations on all 32 wavelength channels simultaneously. Finally, the system executes a joint inference based on the optical intensity information aggregated from all wavelengths, determining the final video classification result. Thus, the PhC-DONN completes multimodal visual classification tasks with only a single optical forward propagation after training. By leveraging the multi-wavelength parallel classification mechanism, this architecture significantly enhances computational throughput while maintaining high classification accuracy in both image and video classification tasks.

### 2.2 Defect design and dynamic control by 2D PhC

**Figure 2:**
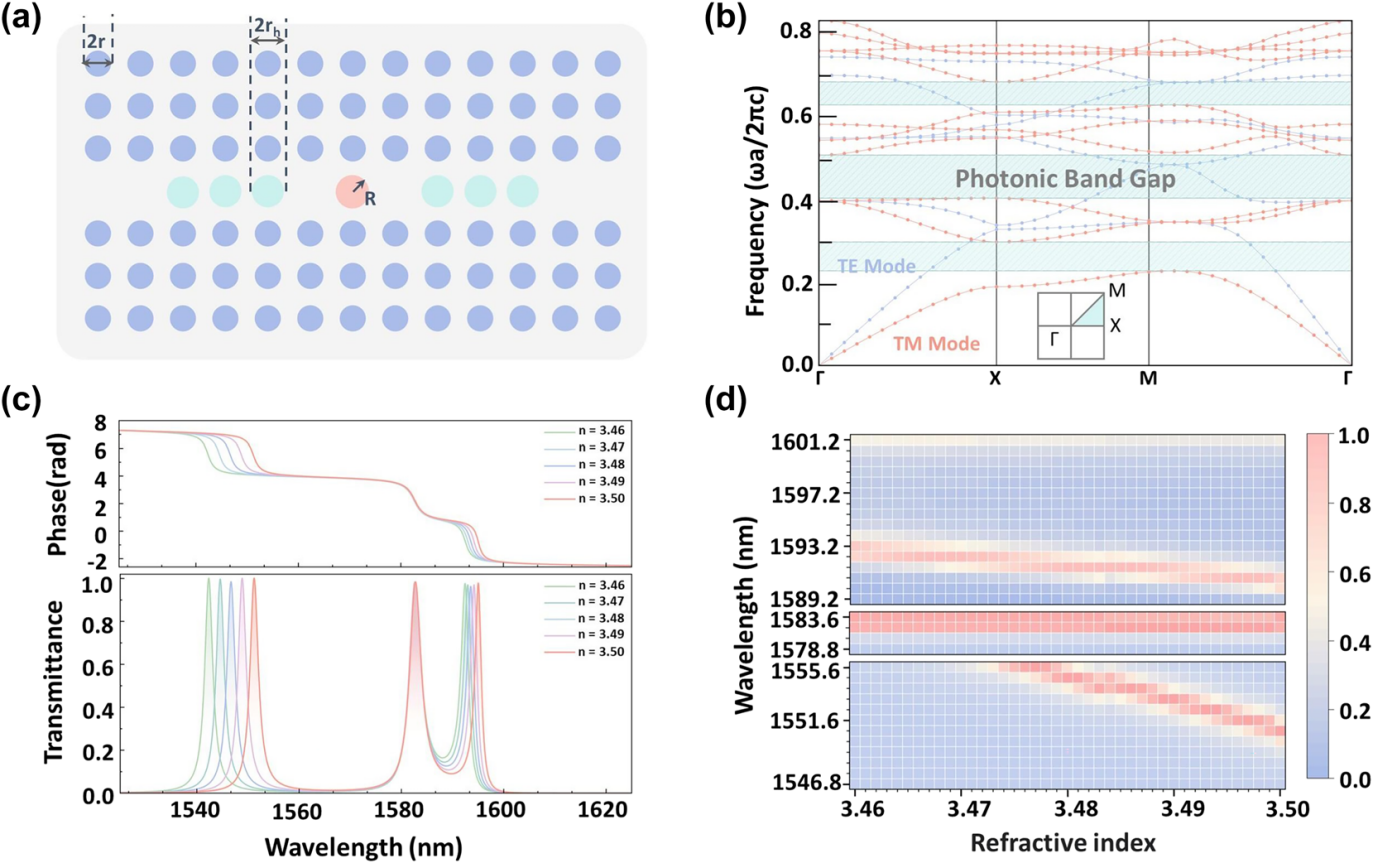
Structure and characteristics of the PhC unit. (a) Schematic representation of the defect structure within the PhC unit. (b) Schematic diagram illustrating the principle of defect-induced resonance, along with the corresponding photonic bandgap. (c) Transmittance spectra demonstrating resonance shifts induced by increasing the refractive index of the central silicon pillar (marked in red). (d) Distribution of transmittance responses across discrete wavelengths, induced by refractive index variations of silicon pillars, with color intensity encoding the amplitude of transmittance.

The 2D square lattice PhC structure is one of the core components of the PhC-DONN, with its primary function being the regulation of input and output optical signals. This structure consists of silicon pillars immersed in an air background, where the refractive index of silicon in the near-infrared band around 1,550 nm being approximately 3.48. The lattice constant of the PhC is set as *a* = 420 nm, and the radius of the silicon pillars is 0.3*a*, as shown in Figure 2(a). To control the optical signal propagation process, this PhC structure introduces a defect in the central region, where a specific cylindrical pillar array is arranged within the defect area to enable the transmission of specific wavelength signals. At the center of the defect region, a silicon pillar with radius *R* = 161.54 nm is placed (marked in red in Figure 2(a)), while three silicon pillars with radius *r*_h_ = 156.15 nm are positioned on each side (marked in green in Figure 2(a)). The designed structure aims to achieve three high transmittance peaks within the 1,530–1,620 nm wavelength range, ensuring independent and efficient transmission of optical signals across multiple channels. To analyze the optical properties of this structure, we calculated its band structure using the finite element method, as shown in Figure 2(b). The results demonstrate a distinct PBG, in which the TE and TM polarized band structures are represented by blue and red curves respectively, and the shaded areas indicate the PBG ranges. For the TM mode, the normalized frequency range of the first PBG is 0.23–0.30*a*/*λ* (corresponding to wavelengths in the range of 1,400 nm < *λ* < 1826 nm), which serves as the basis for our study. In the optical input terminals of the PhC-DONN, we select the TM polarization mode as the primary excitation mode to ensure optimal optical transmission efficiency.

To further investigate the influence of the refractive index on optical signal propagation, Figure 2(c) presents the PhC transmittance and phase response under varying refractive indices. The phase exhibits distinct abrupt shifts covering the full 2π range, demonstrating the defect structure’s capability of complete phase modulation. Under initial conditions (with a refractive index of the central defect silicon pillar, *n* = 3.48), the transmittance curve shows three prominent peaks at 1,546.68 nm, 1,582.66 nm, and 1,593.55 nm within the 1,530–1,620 nm wavelength range. As the refractive index of the central R-pillar increases, these transmittance peaks undergo a redshift, confirming that the refractive index can significantly alter optical transmission characteristics and provide dynamically tunable optical responses for the system. Furthermore, this tunable transmission property enables dynamic adjustment of the network architecture and parameters, substantially enhancing the PhC-DONN’s adaptability across diverse computational tasks.

To more intuitively illustrate the impact of variations in the refractive index on optical transmittance, Figure 2(d) depicts the wavelength-dependent transmittance characteristics under different refractive index conditions using a two-dimensional heatmap. The horizontal axis represents the refractive index of the central silicon pillar *R*, while the vertical axis corresponds to the selected 32 input wavelength groups. Color intensity quantitatively encodes the magnitude of transmittance. These 32 wavelength groups are located within the transmittance peak regions near 1,546.68 nm, 1,582.66 nm, and 1,593.55 nm, ensuring that the study encompasses the system’s optimal transmission window. By adjusting the refractive index of the silicon pillar at the defect center, precise control over the transmittance peaks can be achieved, thereby providing programmability for subsequent computations in photonic neural networks.

## 3 Results

### 3.1 Multi-wavelength classification for static images

**Figure 3:**
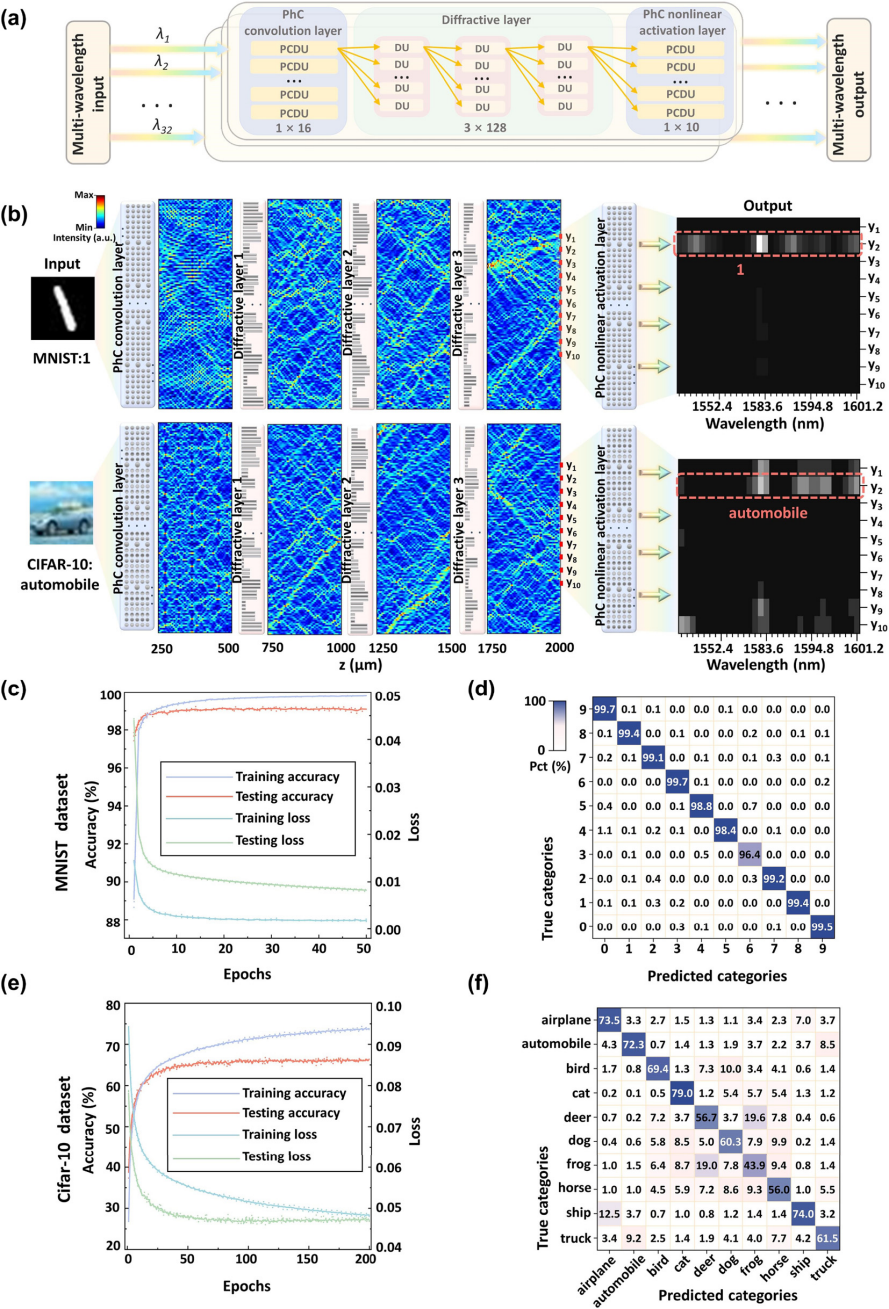
Structure and results of the PhC-DONN for multi-wavelength parallel processing. (a) Multi-wavelength parallel processing architecture, where 32 wavelength-modulated optical signals sequentially pass through the PhC convolutional layer, diffractive layers, and PhC nonlinear activation layer, completing inference through joint multi-wavelength decision-making. (b) Optical transmission process of wavelength-encoded MNIST and CIFAR-10 images within the PhC-DONN architecture. (c) MNIST accuracy-loss convergence curves during training and testing (final test accuracy: 99.09 %). (d) MNIST classification confusion matrix, with color depth representing classification confidence levels. (e) CIFAR-10 accuracy-loss convergence curves (final test accuracy: 66.41 %). (f) CIFAR-10 classification confusion matrix.

The multi-wavelength parallel processing architecture of the PhC-DONN, as illustrated in Figure 3(a), comprises three key components. (1) PhC convolutional layer: The 32 wavelength-modulated optical signals undergo feature extraction using a 1 × 1 convolutional kernel that produces 32 outputs. (2) Diffractive layers: This component includes three layers of diffractive units that implement optical weight computation through silicon groove length-controlled phase modulation. (3) PhC nonlinear activation layer: Achieving nonlinear activation through PhC transmittance responses, mapping the optical signals to 10 output channels. The final classification accuracy is enhanced by superimposing the computational results from all 32 wavelengths. Figure 3(b) illustrates the optical transmission process of wavelength-encoded MNIST and CIFAR-10 images within the PhC-DONN architecture. The left panel displays the input images, the central panel shows the superimposed propagation profiles of 32 wavelength signals across the diffractive layers, and the right panel presents the final output spectra. Training objectives involve mapping input optical signals onto 10 predefined regions (“*y*_1_” – “*y*_10_”) at the output layer, with classification determined by identifying the region with the highest optical intensity at the output layer.

Figure 3(c) and (e) illustrate the accuracy and loss curves during training and testing for the MNIST and CIFAR-10 datasets on the PhC-DONN. For the MNIST dataset, the final test accuracy reaches 99.09 %, and the CIFAR-10 dataset achieves a final test accuracy of 66.41 %. These results demonstrate that the PhC-DONN architecture exhibits adaptability and effectiveness in processing image classification tasks. Although individual wavelength channels may exhibit classification errors during diffractive computations, these errors are compensated through probability superposition across wavelengths at the output stage. This mechanism enhances system fault tolerance by mitigating classification failures caused by single-wavelength errors, highlighting the advantages of joint inference provided by multi-wavelength parallel classification. To illustrate this advantage more intuitively, we present a comparative analysis of the performance of a multi-wavelength joint inference mechanism compared with single-wavelength inference (see Supplementary material S1).

### 3.2 Multi-wavelength inference for dynamic videos

**Figure 4:**
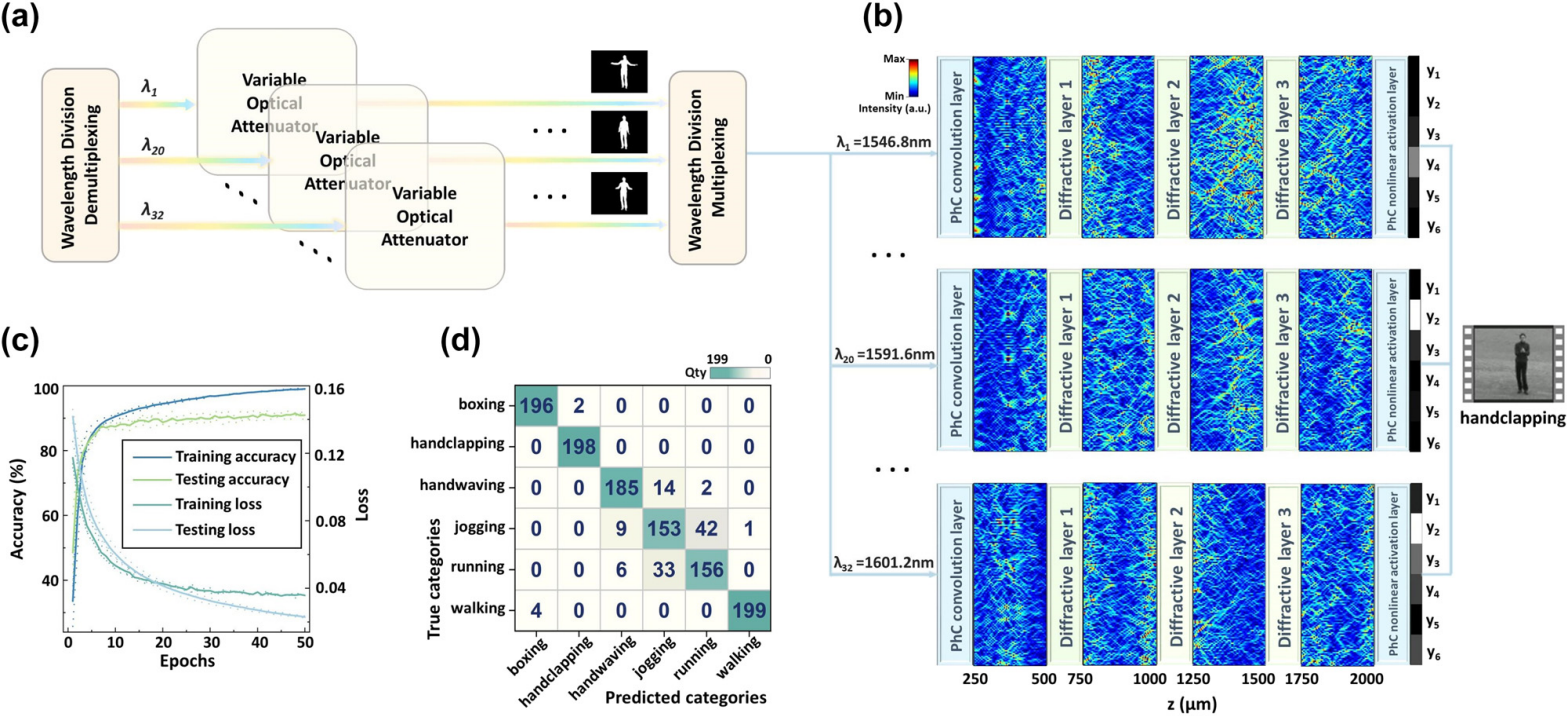
Classification performance of the PhC-DONN on the KTH video dataset. (a) Video sequence preprocessing workflow. (b) Dynamic propagation process of three wavelength signals (1,546.8 nm, 1,591.6 nm, and 1,601.2 nm, corresponding to the 1st, 20th, and 32nd frames of the video sequence, respectively) in the PhC-DONN, with classification inference achieved through multi-wavelength decision-making. (c) Accuracy-loss convergence curves, showing a final test accuracy of 92.25 %. (d) Confusion matrix of classification results on the KTH dataset, where color intensity represents classification confidence levels.

Beyond static images, the PhC-DONN can be extended to a video sequence processing architecture for high-precision human action recognition. As detailed in Figure 4(a), the input video sequence is first temporally decomposed into 32 frames. Each frame is mapped onto 32 distinct wavelength channels to achieve optical encoding of temporal dimensions. Each wavelength channel carries specific frame information through intensity modulation of pixel features. The encoded frames are loaded onto corresponding wavelength channels, and subsequently combined into a single optical path using a wavelength multiplexer (MUX), enabling efficient and parallel frame-level computation. After propagating through the diffractive layers and the nonlinear activation layer, the PhC-DONN completes joint inference of all 32 wavelength signals to determine the final classification result.

The propagation of three selected wavelength signals (*λ*_11_ [1,546.8 nm], *λ*_29_ [1,591.6 nm], and *λ*_30_ [1,601.2 nm]) through the PhC-DONN architecture demonstrates dynamic optical classification for video frame processing, as detailed in Figure 4(b). These optical signals first enter the PhC convolutional layer for initial modulation, then traverse the three diffractive layers that extract spatiotemporal features through phase-controlled diffraction operations. Finally, the signals are mapped onto six predefined regions (*y*_1_ − *y*_6_) at the PhC output layer to determine action categories. The multi-wavelength parallel classification architecture enables joint inference by combining results from all wavelength channels at the output stage. For example, as shown in the right panel of Figure 4(b), the system classifies the video sequence as “handclapping” through a comprehensive analysis of multiple temporal frames rather than through single-frame analysis. This approach enhances the utilization of temporal information and improves classification accuracy compared to conventional frame-by-frame methods.

Figure 4(c) and (d), respectively, depict the accuracy-loss convergence curves and the confusion matrix for the classification of KTH video sequences using the PhC-DONN architecture. The PhC-DONN achieves a classification accuracy of 92.25 % on the KTH video sequences. Compared to traditional frame-by-frame video classification methods, the PhC-DONN simultaneously processes 32 frames within a single optical propagation, significantly improving computational throughput and inference speed. Furthermore, the PhC-DONN maintains high classification accuracy even in complex optical environments due to enhanced noise immunity provided by multi-wavelength signal superposition. Thus, compared to traditional single-wavelength or electronic computing methods, multi-wavelength parallel processing not only improves computational precision and noise immunity but also provides an efficient, low-power optical computing solution.

## 4 Conclusions

This work proposes a two-dimensional photonic crystal-diffractive optical neural network (PhC-DONN) architecture, successfully realizing an on-chip optical parallel classification scheme with multi-wavelength parallel processing. By establishing a wavelength-diffraction joint modulation model, the PhC-DONN overcomes the inherent limitations of traditional on-chip diffractive networks operating in single-wavelength mode, demonstrating exceptional performance in image and video classification tasks.

In the future, the PhC-DONN can be expanded to address complex scenarios, such as 100-class classification tasks and high-resolution image classification, and even potential applications in real-time multi-object tracking for autonomous driving (utilizing wavelength-encoded spatial coordinates) and 3D reconstruction of pathological slices (employing wavelength dimensions to map depth information). These two application domains demonstrate the same underlying neural-network principles as the PhC-DONN’s approach to dynamic video recognition. For structural performance optimization, the PhC-DONN can achieve global optimization of the multi-wavelength modulation matrix through inverse design and multi-objective optimization algorithms, thereby enhancing computational performance. Recent advances in photonic inverse-design algorithms suggest that joint optimization of device geometry and wavelength routing can further amplify throughput gains. Furthermore, the PhC-DONN can be integrated with photonic devices such as micro-ring resonators (MRRs), Mach–Zehnder interferometers (MZIs), and photonic crystal modulators (PCMs) to construct terabits per second (Tbps) optical computing chips. While our current structure utilizes only 32 discrete wavelength channels, emerging frequency-comb sources promise scalable channel counts in the near future. We believe that the multi-wavelength parallel classification mechanism of the PhC-DONN will propel the advancement of optical neural networks and establish a new paradigm for high-efficiency optical artificial intelligence processors.

## Supplementary Material

Supplementary Material Details
