# Supplementary material for: Multi-wavelength diffractive optical neural network integrated with 2D photonic crystals for joint optical classification
Source: Nanophotonics. 2025 Jul 8;14(17):2891–9. doi: 10.1515/nanoph-2025-0168 (PMC12397731; doi:10.1515/nanoph-2025-0168)
Supplement: Supplementary file 1 — Supplementary Material Details [file j_nanoph-2025-0168_suppl_001.docx]

multi-wavelength diffractive optical neural network integrated with 2d photonic crystals for joint optical classification: supplemental document

1. **Comparison of classification performance between multi-wavelength and single-wavelength models**

As shown in Fig. S1(a)-(d), the red curves represent the results based on the joint inference of 32 sets of wavelengths, and the blue curves represent the statistical results of the single-wavelength independent inference. In the MNIST test set, the maximum accuracy of the multi-wavelength model reaches 99.09%, which is approximately 0.69% higher than the single-wavelength model's average accuracy of 98.40%. The single-wavelength model demonstrates a maximum accuracy of 99.04%, a minimum of 95.57%, and a sample variance of 0.3535, indicating substantial performance fluctuations across individual channels. The multi-wavelength mechanism effectively mitigates such instability caused by partial bias. For the more complex CIFAR-10 task, the multi-wavelength model stabilizes at 66.41% accuracy after 200 epochs, surpassing the single-wavelength model's average (62.23%) by 4.18%. The single-wavelength results exhibit higher variability, with maximum and minimum accuracies of 64.58% and 46.88%, respectively, and a sample variance of 18.088. The multi-wavelength joint inference achieves efficient optical signal propagation by independently regulating the transmittance of 32 wavelength channels through the PhC convolutional layer, combined with the diffraction layer's fixed-weight linear operations. As shown in Fig. S1(a), the multi-wavelength model attains 98.92% accuracy on the MNIST test set at 10 epochs, whereas the single-wavelength model requires 30 epochs to reach a similar level (98.90%), demonstrating a 3-fold acceleration in convergence. This rapid convergence not shortens training cycles but also reduces dynamic energy consumption in optical device regulation, offering critical support for energy-efficient photonic hardware.


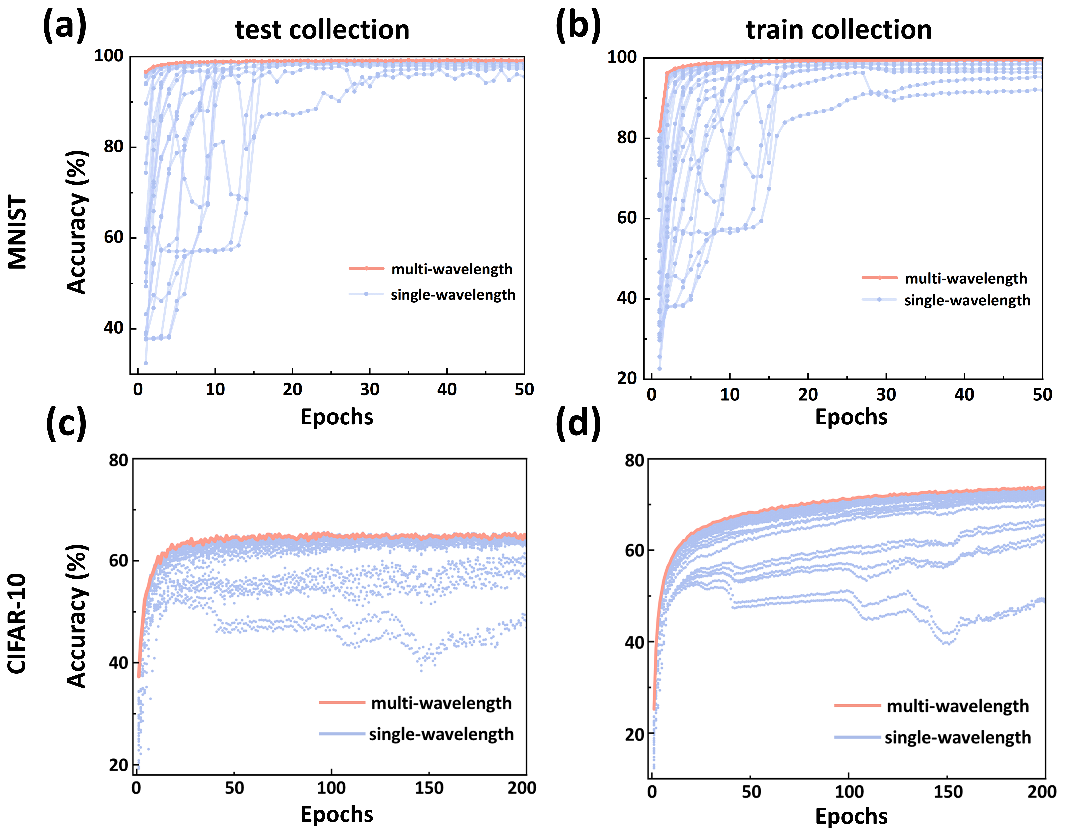


FIG. S1. Performance comparison between multi-wavelength joint inference and single-wavelength independent inference on MNIST and CIFAR-10 datasets. (a) Comparison of the accuracy on the MNIST test set: the red curve is the result of multi-wavelength joint inference, and the blue curve is the result of 32 sets of single-wavelength independent inference. (b) Trend of the accuracy on the MNIST training set. (c) Comparison of the accuracy on the CIFAR-10 test set (multi-wavelength vs. single-wavelength). (d) Trend of the accuracy on the CIFAR-10 training set.

In our PhC-DONN design, we selected 32 discrete wavelength channels based on a balance of spectral coverage, weight efficacy, and parallelism. As shown in Fig. 2(c), the device’s transmission spectrum exhibits three distinct peaks spanning 1542–1555 nm, 1577–1587 nm, and 1588–1603 nm. By choosing 32 channels with 0.8 nm spacing—determined by the bandwidth and stability constraints of our current optical frequency comb, we ensure that each channel coincides with a transmission peak and yield nonzero modulation.

From the standpoint of weight adjustment, due to the transmission spectrum of the PhC structure，an overly large number of channels would introduce many lines with near-zero transmittance, which do not contribute to the network’s functionality. Conversely, too few channels would limit convolutional parallelism and reduce overall throughput. 32 channels represent an optimal trade-off: they densely populate the high-transmission regions while maintaining sufficient parallel processing capability for moderate-complexity tasks. In addition, designing photonic-crystal elements with lower quality factors will broaden each resonance’s bandwidth and expand tunable ranges, further supporting channel count expansion.

1. **Forward propagation model for PhC-DONN neural network**

Conventional deep neural networks rely on forward propagation, backward propagation, and gradient descent algorithms to perform electronic computation by continuously adjusting the weights of electronic neurons. The light diffraction that occurs during propagation is very similar to the way neurons are connected in deep neural networks. Based on Rayleigh-Sommerfeld diffraction, each diffracting unit/neuron can be viewed as a coherent superposition of light propagating from each diffracting unit/neuron in the pre-diffraction layer. It can also be viewed as a source of secondary waves fully connected to the subsequent layer. The equation of light propagation between diffraction layers is as follows:

$$\begin{aligned} \omega_{i}^{l}\left( x,z \right)=\frac{z-z^{i}}{r^{2}}\left( \frac{1}{2\pi r}+\frac{1}{j\lambda} \right)\exp\left( \frac{j2\pi r}{\lambda} \right),\#(1)\# \end{aligned}$$

where $\omega_{i}^{l}\left( x,z \right)$ is a complex-valued field located in the ith diffraction unit at coordinates $(x_{i},z_{i})$ in the lth layer, and the complex-valued optical field $\left( x,z \right)$ is propagated to the diffraction unit located at $\left( x,z \right)$ in the l+1’th layer using the wavelength λ as the wave source. The propagation distance is defined by the following equation:

$$\begin{aligned} r=\sqrt{\left( x-x_{i} \right)^{2}+\left( z-z_{i} \right)^{2}},\#(2) \end{aligned}$$

where $j^{2}=-1$ is an imaginary unit. The light field function $u_{i}^{l}$ for the ith neuron in layer l can be expressed as:

$$\begin{aligned} u_{i}^{l}\left( x_{i},z_{i} \right)=\sum_{j\in N} u_{j}^{l-1}\left( x_{j},z_{j} \right)\cdot t^{l}\left( x_{i},z_{i} \right)\cdot\omega_{i}^{l-1}\left( x_{i},z_{i} \right),\#(3) \end{aligned}$$

In the optical diffraction network, the complex-valued modulation of the optical field by the l’th diffraction layer is described by the function $t^{l}\left( x_{i},z_{i} \right)$, whose expression is $t^{l}\left( x_{i},z_{i} \right)=a^{l}\left( x_{i},z_{i} \right)\cdot exp[jФ^{l}\left( x_{i},z_{i} \right)]$, in which $a^{l}$ and $Ф^{l}$ denote the amplitude modulation coefficient and the phase modulation coefficient, respectively, both of which are trainable parameters in the diffraction network. The values of the amplitude coefficient $a^{l}$ range from 0 to 1, and the values of the phase coefficient $Ф^{l}$ range from 0 to 2π, which corresponds to the full-phase period modulation of the light wave. The parameter n represents the set of all pixels in the previous diffraction layer, whose light fields are transferred to the current layer through the above modulation function to realize the linear transformation and nonlinear activation of the optical signal.

In order to reduce the computational complexity of the traditional diffractive neural network based on the Rayleigh-Sommerfeld formula, the Fresnel scalar diffraction theory is used in this study to construct the forward propagation model of diffractive neural network. The theory can replace the Rayleigh-Sommerfeld formula when the layer spacing satisfies the evening axis condition. The complex amplitude $u_{i}^{l}\left( x_{i} \right)$of the ith neuron in the lth layer is realized in the null-frequency domain by the fast Fourier transform (FFT) and its inverse transform (IFFT), and its expression is:

$$\begin{aligned} u_{i}^{l}\left( x_{i} \right)=F^{-1}\left\{ F\left[ u_{i}^{l-1}\left( x_{i} \right)\cdot t^{l-1}\left( x_{i} \right) \right]\cdot H\left( f_{x} \right) \right\},\#(4) \end{aligned}$$

$$\begin{aligned} H\left( f_{x} \right)=\exp\left[ jk\left( z-z_{i} \right) \right]\cdot\exp\left[ -j\lambda\pi\left( z-z_{i} \right)\cdot f_{x}^{2} \right],\#(5) \end{aligned}$$

where $F$ and $F^{-1}$ denote the fast Fourier transform and its inverse transform, respectively, $t^{l-1}$ is the complex transmittance modulation function of the diffraction unit of the l-1’th layer, and $H\left( f_{x} \right)$ is the frequency-domain transfer function, which describes the propagation characteristics of the beam in free space. Where *k=2π/λ* is the wave number, λ is the wavelength, and $z_{i}$ is the propagation distance of the ith layer. The model reduces the propagation integral to a frequency-domain multiplication operation by FFT, which significantly improves the computational efficiency of the diffraction neural network.

Figures S2(a)-(b) present the finite element method (FEM) simulation results for optical field propagation. Figure S2(a) illustrates the light intensity distribution across a 100 μm × 100 μm region with a 30 μm-diameter broadband light source, while Figure S2(b) displays the corresponding phase modulation characteristics. Figures S2(c)-(d) demonstrate the numerical solutions obtained from our derived forward propagation equations, implemented in Python. The optical field intensity distribution in Figure S2(c) demonstrates excellent agreement with FEM simulations, confirming the validity of our propagation equations for large-scale diffractive network. Figure S2(d) further verifies the phase calculation accuracy through quantitative comparison with COMSOL multiphysics simulations, establishing the reliability of our computational framework.

All PhC-DONN models in this work were constructed and trained based on PyTorch 2.1.2, implemented within a Python 3.9 computational framework. The simulations were executed on a 64-bit workstation equipped with an Intel Core i9-10940k CPU, NVIDIA GeForce RTX 4080 Ti GPU, and 256GB DDR4 system memory. Training convergence on the CIFAR-10 dataset required 200 epochs with a total computation time of approximately five hours.


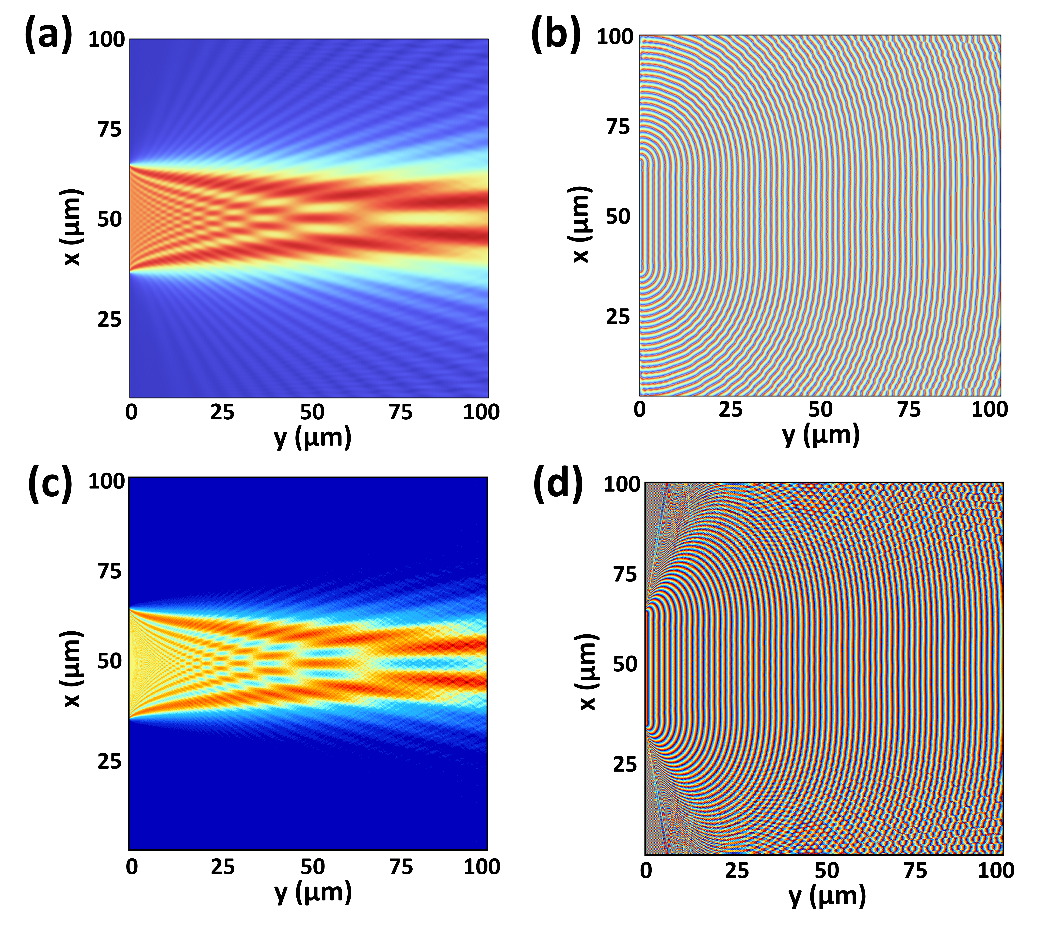


FIG. S2. Simulation and code verification of light propagation and phase distribution. (a) Intensity distribution of the light field in a 100 μm × 100 μm region, setting the width of the incident light source to 30 μm. (b) Phase modulation distribution in the corresponding region. (c) Reconstruction results of the light propagation code based on Rayleigh-Sommerfeld diffraction. (d) Effect of the phase distribution code generation.

1. **Feasibility analysis of structural fabrication**
   1. Design and characteristic analysis of TiN Microheater

There are several factors to consider when choosing a material for use as a heater. Based on properties such as low resistivity, oxidation resistance, and high melting point, we believe that TiN micro-heater can be selected. A self-supporting TiN/SiO2 structure was obtained due to the substrate bias in SiO2 film deposition and stress relaxation through post-annealing. For heater characterization, the minimum power dissipation of the TiN miniature heater was about 70 mW[1]. The TiN heating layer was higher than the Si columns, ensuring a balance between heating efficiency and metal absorption losses. When a voltage is applied to the heater, it transfers heat, which subsequently causes the temperature of the red silicon column to change, altering its refractive index. This modulation of the refractive index enables the control of light transmission in the medium, thereby forming a PhC weight library. Despite some difficulties, this type of heating is feasible, and we can fabricate the micro-heaters using a single exposure operation and two standard wet etching operations.

- 1. PhC structure fabrication error analysis

PhCs can be fabricated by thin-film deposition followed by high-resolution pattern transfer techniques, such as electron-beam lithography (EBL), 193-nm deep-UV lithography, holographic lithography, and nano-imprint; the patterns are then transferred by either dry (ICP-RIE) or wet etching[2]. In practice, EBL is the most common approach for research-grade devices and yields a post-lithography critical-dimension (CD) variation of roughly ± 5 nm. When the pattern is etched into the 220-nm Si device layer using ICP-RIE, an additional CD spread of ± 2–5 nm is typical[3,4]. Consequently, we adopt a conservative ± 5 nm fabrication error in the simulations, applied to both the lattice constant a and the column radius r, to represent the combined worst-case deviation.

We first quantified isolated perturbations: A randomly selected Si column (green highlight, Fig. S3a) underwent radius variations of ±5 nm across 15 independent simulations. Separately, positional displacements (5 nm along x- or y-axes) were applied to another randomly chosen column (red highlight, Fig. S3b) in 15 additional trials. The ensemble-averaged transmission spectra from these 30 simulations are compared against the ideal spectrum in Fig. S3c.

To assess cumulative error impacts, we further simulated complex multi-column perturbations: Ten randomly selected columns simultaneously experienced (i) radius changes (±5 nm), (ii) positional shifts (5 nm), or (iii) combined radius/position alterations (Fig. S3d). Resultant transmission spectra are overlaid with the ideal case in Fig. S3e. These simulations show that, although fabrication errors do alter the transmittance spectrum, the impact is modest; within the practical design tolerances, the spectral deviations remain well within an acceptable range.


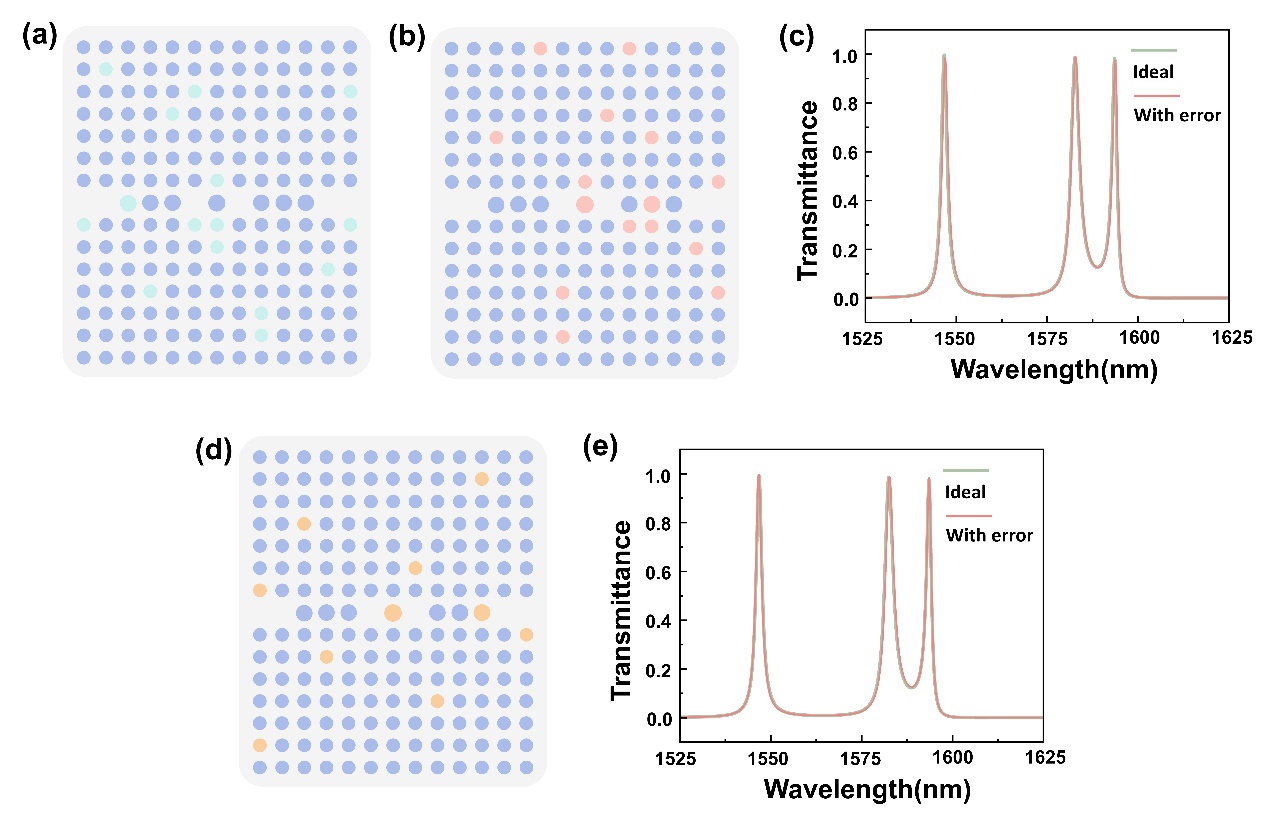


FIG. S3. (a)Single-column radius perturbation: one column is randomly selected and its radius is changed by ± 5 nm (green). (b)Single-column displacement: one column is randomly selected and shifted by ± 5 nm in x or y (red). (c)Transmittance spectra comparing the ideal design (green) with the average of 30 single-column-error simulations (red). (d)Composite error: ten columns are simultaneously given random ± 5 nm radius and/or position errors (orange). (e)Transmittance spectra comparing the ideal design (green) with the composite-error case (red).

- 1. Thermal tuning stability of miniature micro-heater

In this work, we integrate a self-supporting TiN/SiO₂ micro-heater atop the Si column, with a minimum dissipation power of approximately 70 mW[5]. The heater layer is positioned slightly above the column to balance heating efficiency and metal absorption loss. The thermal tuning stability of on-chip micro-heaters is typically characterized by three metrics:

Static Power Stability: Under DC bias, when the TiN heater is held at a constant voltage, its power dissipation varies by less than 0.1 mW over ten minutes. Because the silicon substrate and nanostructure exhibit excellent thermal stability, this electrical power stability corresponds to a negligible drift in the column’s temperature. Consequently, the center wavelength and phase of the transmission spectrum remain effectively constant.

Temperature–Transmission Response and Time Constant: Both simulations and experiments show that, as the TiN heater is driven from 0 mW to 70 mW (the temperature rise of the Si column is approximately 143 K)[6,7], the majority of the heating or cooling process completes within roughly 5.6 μs. This rapid thermal response ensures that, at a MHz-level update rate, the Si column reaches its steady-state temperature almost immediately. Any subsequent minor power fluctuations (e.g., from supply noise or ambient temperature variations) only affect the transmission spectrum for a few microseconds before being corrected, preventing cumulative long-term drift.

Thermal Crosstalk: When the TiN heater dissipates about 25 mW, the steady-state temperature rise at the center of an adjacent, unheated Si column (spaced 420 nm apart) is only ~2.8 K. This corresponds to an induced refractive index change of roughly 5×10⁻⁴. Because each Si column in the photonic-crystal unit is surrounded by air—most of the heat dissipates vertically, and lateral spreading is limited. As a result, actual thermal crosstalk is generally even lower than this estimate. Hence, without any additional isolation, the refractive-index perturbation and transmission-spectrum shift induced in neighboring Si columns by heating a single Si column are negligible and well below the network’s tolerable error threshold.

In summary, using a TiN/SiO₂ micro-heater atop a Si column—even in a densely packed photonic-crystal array—yields extremely low thermal crosstalk and virtually no impact on the transmission spectrum. This satisfies the stringent thermal tuning stability requirements for constructing a programmable phase-delay library. Despite certain fabrication challenges, this heating approach is feasible.

**4. Light utilisation efficiency in the system**

In our PhC-DONN system, the overall light-utilization efficiency can be estimated by combining the losses in the PhC convolutional units and the diffractive layers.

For the 2D PhC waveguides, propagation loss is highly sensitive to the choice of materials, structural design, and fabrication precision. On a silicon-on-insulator (SOI) platform, the average loss is approximately 0.8 ± 0.2 dB/mm[8,9]. In our design, each PhC unit has a lattice constant of a = 420 nm, a Si column radius of r = 120 nm, and spans 13 columns laterally, giving a unit-cell length of L = 13 × a = 5.7 μm. Over this length, the theoretical transmission loss corresponds to 0.026%–0.105%. To account for additional facet-coupling losses and fabrication variations encountered in experiment, we conservatively budget a 1% loss per PhC unit—i.e. a transmission efficiency of 99%.

The dominant loss in the DONN structure stems from Fresnel reflections at each diffractive interface. Assuming normal incidence between Si (n_1_=3.48) and SiO_2_ (n_2_=1.44) at 1550 nm, the per-layer transmittance is

$$\begin{aligned} T=1-\frac{\left( n_{1}-n_{2} \right)^{2}}{\left( n_{1}+n_{2} \right)^{2}}\approx82.8\%,\#\left( 6 \right) \end{aligned}$$

so three successive diffractive layers pass about 0.828^3^ ≈ 56.8% of the light.

Multiplying the PhC convolutional efficiency (99%) by the three-layer diffractive network (56.8%) and including an additional PhC-based nonlinear activation stage, we arrive at an overall theoretical light-utilization efficiency of approximately 55.6%. In experimental tests, total losses will exceed this ideal value; however, by reducing the number of diffractive layers or optimizing interface coatings, these losses can be mitigated. As we are dealing with an optical computational network, we only need the detected the effective optical signal against noise to meet the minimal requirements for computation.

**5. Silicon Slot-Based Neuronal Phase Modulator Design**

In our integrated PhC-DONN architecture, on-chip electromagnetic wave propagation is modeled using analytical expressions, with network structure parameters pre-trained through forward propagation and error backpropagation algorithms. To ensure reliable physical mapping of the pre-trained phase values, we implement silicon slots filled with silicon dioxide (SSSD) as neuronal phase modulators. The length of each SSSD unit is determined by the phase delay requirement through the expression:

$$\begin{aligned} L_{slot-i}=\frac{{}_{i}}{\left( n_{eff}-n_{slab} \right)\cdot k_{0}},\#\left( 7 \right) \end{aligned}$$

where $L_{slot-i}$ denotes the SSSD length in the *i*-th modulation group, $n_{eff}$​ represents the effective refractive index of the SiO₂-filled slot waveguide region, $n_{slab}$​ is the effective index of the slab waveguide, *k*_0_​=2*π*/*λ* is the vacuum wavenumber, and ${}_{i}$​ corresponds to the target phase delay for the *i*-th silicon dioxide slot. This approach enables precise translation of numerically optimized phase values to manufacturable photonic structures.

**6. Insertion loss of elements**

Insertion Loss of the PhC Structure:

The insertion loss of the 2D PhC waveguide mainly includes propagation loss and facet coupling loss. On a SOI platform, the propagation loss of 2D PhC waveguides is typically in the range of 0.5–2 dB/mm[8,9]. In our design, each PhC unit has a lattice constant a=420 nm, a Si column radius r=120 nm, and spans 13 columns laterally, resulting in a unit-cell length L=13×a=5.7μm. Over this length, the theoretical propagation loss is:

$$\begin{aligned} 0.5\frac{dB}{mm}\times0.0057mm\approx0.00285dB,\#\left( 8 \right) \end{aligned}$$

and in the worst case (2.0 dB/mm), the loss is 0.0114 dB. To account for additional facet-coupling losses and fabrication variations encountered during experiments, we conservatively allocate 0.044 dB insertion loss per PhC unit (corresponding to 1% loss, since -10log10(0.99) = 0.044 dB). Similarly, the photonic PhC nonlinear activation layer also has an insertion loss of 0.044 dB.

Facet coupling loss primarily arises because the output of the PhC waveguide is typically a compact Bloch mode, whereas the diffractive network requires normal or near-plane wave incidence. Without additional mode-shaping techniques (such as tapered waveguides, mode converters, or microlens arrays), this mode mismatch generates extra coupling loss, typically around 0.5 dB. Thus, for the design parameters of lattice constant a = 420 nm and unit length of 5.7 µm, the propagation loss is negligible, and the dominant loss comes from facet coupling, with a total insertion loss of approximately 1 dB.

Insertion Loss of the DONN:

The loss in the diffractive network mainly originates from Fresnel reflection losses at each Si/SiO₂ interface. Given the refractive indices of silicon $n_{\mathrm{Si}}$ = 3.48 and silicon dioxide $n_{\mathrm{SiO}_{2}}$ = 1.44, the per-layer transmittance is

$$\begin{aligned} T=1-\frac{\left( n_{Si}-n_{{SiO}_{2}} \right)^{2}}{\left( n_{Si}+n_{{SiO}_{2}} \right)^{2}}\approx82.8\%,\#\left( 9 \right) \end{aligned}$$

which corresponds to an insertion loss of approximately 0.82 dB per layer. Since we use three consecutive diffractive layers, the total insertion loss from the diffractive network is 2.46 dB.

In total, considering the PhC unit ( 0.6–1 dB) and the diffractive network (2.46 dB), the combined insertion loss is approximately 3–3.5 dB. The sensitivity of the PhC-DONN to insertion loss depends on the specific task’s signal-to-noise ratio (SNR) requirements and the detection sensitivity. In general, if the insertion loss is too high, it can lead to a decrease in the SNR and classification performance. However, within the range of 3–4 dB, our optical computational network can still reliably maintain high performance, striking a good balance between SNR and computational efficiency.

**References**

1. M. Jacques et al., "Optimization of thermo-optic phase-shifter design and mitigation of thermal crosstalk on the SOI platform," Opt. Express, vol. 27, no. 8, pp. 10456–10471, 2019.
2. L. Y. Jiang et al., "Design and fabrication of rod-type two-dimensional PhC slabs with large high-order bandgaps in near-infrared wavelengths," Opt. Lett., vol. 37, no. 9, pp. 1424–1426, 2012.
3. X. Zhao et al., "Dependency analysis of line edge roughness in electron-beam lithography," Microelectron. Eng., vol. 133, pp. 78–87, 2015.
4. S. K. Selvaraja et al., "Fabrication of Photonic Wire and Crystal Circuits in Silicon-on-Insulator Using 193-nm Optical Lithography," J. Lightw. Technol., vol. 27, no. 18, pp. 4076–4083, 2009.
5. H. Ito, Y. Kawamata, and H. Nikkuni, "Fabrication and evaluation of suspended Micro-heater using TiN," Electr. Eng. Jpn., vol. 214, no. 2, pp. 1–6, 2021;
6. N. A. Nobile et al., "Time-resolved temperature mapping leveraging the strong thermo-optic effect in phase-change materials," ACS Photonics, vol. 10, no. 10, pp. 3576–3585, 2023.
7. M. A. Jithin et al., "Development of titanium nitride thin film micro-heaters using laser micromachining," Vacuum, vol. 197, p. 110795, 2022.
8. M. Notomi et al., "Waveguides, resonators and their coupled elements in photonic crystalPhC slabs," Opt. Express, vol. 12, pp. 1551–1561, 2004.
9. D. Gerace and L. C. Andreani, "Low-loss guided modes in photonic crystalPhC waveguides," Opt. Express, vol. 13, pp. 4939–4951, 2005.
